# Interstate Variation in Modifiable Risk Factors and Cardiovascular Mortality in the United States

**DOI:** 10.1371/journal.pone.0101531

**Published:** 2014-07-08

**Authors:** Shivani A. Patel, K. M. Venkat Narayan, Mohammed K. Ali, Neil K. Mehta

**Affiliations:** Hubert Department of Global Health, Rollins School of Public Health, Emory University, Atlanta, Georgia, United States of America; Arizona State University, United States of America

## Abstract

**Objective:**

We investigated the role of state-level differences in modifiable cardiovascular (CV) risk factors in contributing to state disparities in cardiovascular mortality rates in the US.

**Methods:**

Adults aged 45–74 in 2010 were examined. We constructed a CV risk index summarizing state-level exposure to current smoking, obesity, physical inactivity, alcohol abstinence, hypertension, elevated cholesterol, and diabetes using the Behavioral Risk Factor Surveillance System. Outcomes were cardiovascular, coronary heart disease, and stroke mortality. Linear regression was used to estimate associations between the CV risk index and mortality outcomes. Models accounted for state-level socioeconomic characteristics and other potential confounders.

**Results:**

Risk factors were highly correlated at the state-level (Cronbach's alpha 0.85 (men) and 0.92 (women). Each +1SD difference in the cardiovascular risk index was associated with higher adjusted cardiovascular mortality rates by 41.0 (95%CI = 26.3, 55.7) and 33.3 (95%CI = 24.4, 42.2) deaths per 100,000 for men and women, respectively. The index accounted for 8% (men) and 11% (women) of the variation in state-level cardiovascular mortality. Comparable associations were also observed for coronary heart disease and stroke mortality.

**Conclusions:**

CV risk factors were highly correlated at the state-level and were independently associated with state CV mortality, suggesting the utility of generalized CV risk reduction.

## Introduction

The United States has achieved significant progress in reducing mortality and improving health over the past few decades, including an overall reduction in cardiovascular (CV) disease mortality [1]. The country's standing in population health, however, lags behind most high-income countries [2]. In addition to America's poor ranking in health and longevity internationally, there are considerable geographic health and mortality disparities within the US. Coronary heart disease (CHD) and stroke, among the leading causes of years of life lost in the US [2], vary substantially across US states [3], [4], such that CHD prevalence, for example, among adults in Kentucky (8.2%) was over double that in Hawaii (3.7%) in 2010 [5].

A large body of evidence at the individual-level has identified a number of modifiable behavioral and health factors to be critical for reducing CV-related morbidity and mortality [6], [7]. Previous studies showed that, like adverse CV outcomes, these risk factors are unevenly distributed across US states [4], [8], [9]. In the early 1990s, Byers et al. further reported a positive correlation between the prevalence of leading modifiable risk factors—tobacco use, excess body weight, physical inactivity, hypertension, elevated cholesterol, diabetes, and alcohol abstinence—and CV mortality across US states [10]. In the two decades since this earlier work, there have been considerable changes in the distribution of risk factors nationally and improvements in medical treatments targeting CV disease [11].

The extent to which state-level CV mortality differentials continue to be associated with risk factor distribution and whether these state mortality differentials are driven by modifiable risk factor distributions as opposed to state socioeconomic and demographic compositional factors remains unclear. Contemporary understanding of the relation between modifiable risk factors and CV mortality at the state-level may inform health policy, as states are both at the frontline for effective action to prevent and control disease and hold much public health authority. Furthermore, studying the contribution of modifiable risk factors in explaining state-level differences in CV mortality may aid in quantifying expected effects of risk factor reduction on state CV mortality disparities.

Using mortality data from 2010, we investigated the hypothesis that CV risk factors collectively continue to contribute to CV mortality differences across US states by examining (1) associations between an aggregate CV risk index with CV mortality rates and (2) the proportion of CV mortality variation independently attributable to the CV risk index across US states and DC.

## Materials and Methods

### Data sources

We used CV mortality data from the National Center for Health Statistics (NCHS) for adults aged 45–74 years in all 50 US states and Washington DC (DC) in 2010. State-level data on modifiable risk factors corresponding to this birth cohort were obtained from the 2005 survey of the Behavioral Risk Factor Surveillance System (BRFSS), an annual nationally-representative cross-sectional telephone survey of the US population. The five year lag was used to account for the potential delay between exposure to a risk factor (e.g., smoking) and the development of disease. We compiled risk factor data from 196,849 respondents aged 40–69 surveyed by BRFSS in 2005. A unique strength of the BRFSS is that, with weighting, it provides prevalence data that are representative at the state level. We found that 2001 and 2010 state-level risk factor prevalence estimates in this birth cohort were highly correlated with 2005 estimates, and analyses using risk factor data from those years were consistent with findings reported here.

### Measures and Definitions

We used the International Statistical Classification of Diseases and Related Health Problems 10th Revision (ICD-10) to classify deaths due to CV disease (ICD-10 codes I00-I99) and its major components, CHD (ICD-10 I20-I25) and stroke (ICD-10 I60-I69). Mortality rates for each state were sex- and age-standardized to the 2010 US national population. The age groups used were 45–54, 55–64, and 65–74.

Risk factors were self-reported and defined as follows: smoking (current smoking), obesity (body mass index [BMI] ≥30 kg/m^2^; BMI computed from respondent-reported height and weight data), physical inactivity (no leisure time physical activity in the past month), alcohol abstinence (no alcohol consumption in the past month), hypertension (ever been told by a health professional that blood pressure was high), elevated cholesterol (ever been told by a health professional that cholesterol was high), and diabetes (ever been told by a health professional that he or she had diabetes). These indicators correspond to established risks to ideal CV health [6] and were comparable to those used in previous state-level analyses [10]. State-level prevalence estimates were weighted using the BRFSS survey weights and sex- and age-standardized to the 2010 US national population.

We found that the risk factors were highly correlated at the state-level in our preliminary analysis. As a summary measure of state-level exposure to CV risk factors, we constructed a composite CV risk index using principal components analysis (PCA). This index allowed us to examine the associations of all risk factors simultaneously with mortality. PCA is a data reduction technique that produces uncorrelated weighted linear combinations (“principal components”) of potentially correlated variables [12]. The state-level prevalence of each risk factor was standardized to have mean = 0 and SD = 1 and treated as a linear predictor in the PCA. State CV risk indices were computed separately for men and women. Two principal components were extracted for men and one for women; following convention, we used the first principal component (i.e., the component capturing the most variation among indicators) as the CV risk index. The final sex-specific CV risk index was a continuous score, ranging from −1.9 to 2.1 for men and −1.6 to 2.5 for women, standardized to have a mean = 0 and SD = 1 across states. Component loadings and standardized scoring coefficients from the PCA are reported in Tables S1 and S2 in File S1. Because the CV risk index was standardized, one unit of the index was equivalent to one standard deviation.

Several covariates that may be related to both risk factor prevalence and CV mortality were taken into consideration [13]–[15]. These data were from the county-level Census Small Area Income & Poverty Estimates (SAIPE), obtained from the 2006 County-level BRFSS data set or directly from the US Census. Economic indicators included median income in 2010 (1000 USD) and the percent of the population that was living below the poverty line in 2004. Demographic indicators included the proportion of the population that was Hispanic and was black in 2004. Low education was defined as more than 25% of the population ages 25 to 64 lacking a high school diploma or GED in 2000, and low employment was defined as less than 65% of the population ages 21 to 64 being employed in 2000. The number of medical specialists per 100,000 included cardiovascular specialists in addition to other specialists, in 2005. The percent of the population that was insured was computed from the BRFSS in 2005. For state-level analyses, these covariates were aggregated up to the state-level by weighting each county's estimate by its population size.

### Analytic Approach

We described the distribution, including relative standard deviation, of risk factor prevalence and cause-specific mortality for all states. The relative standard deviation (RSD), defined as 100× SD/mean, is dimensionless and allows for comparison of variability across risk factors. To assess construct validity, we examined the principal component loadings (Pearson's correlation coefficients) of each risk factor prevalence with the composite CV index. To further assist in the interpretation of the CV risk index, the mean state-level prevalence for each risk factor was computed by quintiles of the index. Pearson's correlations were also used to examine the correspondence between each risk factor prevalence and cause-specific mortality pair. Two sets of linear regression models were estimated. The first included only the CV risk index as a continuous variable as the exposure (unadjusted model). The second (adjusted model) included adjustments for potential confounding covariates. To quantify the proportion of the variance in mortality explained by the CV risk index in the state-level models, we computed the R^2^ for the unadjusted models and the semi-partial R^2^ for the adjusted models. The semi-partial R^2^ for the CV risk index may be interpreted as the proportion of variance in state mortality explained by the CV index after the index was adjusted for confounders.

To strengthen inference, we took three additional steps. First, we conducted the analysis after excluding stroke belt states to reduce the possibility that observed associations were due exclusively to differential risk between stroke and non-stroke belt states. Following Howard et al [4], the stroke-belt was defined as NC, SC, GA, AL, MS, TN, AR, LO, and non-stroke belt states were all other states and Washington DC. Second, we examined transport accident mortality (ICD-10 codes V01-V99) and all-cause mortality among infants (under 1 year of age; mortality is defined as number of deaths per 1000 live births) as “negative controls” [16] based on the assumption that these deaths should not be causally associated with lifestyle-related risk factors or biologic CV risks. Third, we did a parallel county-level analysis to assess whether our state-level findings were due to high risk counties. We estimated models for (1) the entire subset of counties that were sampled by the BRFSS in 2005 and reported at least 20 CV deaths in 2005–2010, (2) the subset of counties below the 90^th^ percentile of the CV risk index, and (3) the subset of counties below the 90^th^ percentile of CV mortality rates in 2005–2010.

All exposures and outcomes were assessed for normality and no substantial departures were detected (skewness and kurtosis ranged from −1 to 1 for each variable). Linearity of the association between the CV index and mortality rates was visually assessed (Figure S1). Statistical analyses were performed in SAS 9.2 (SAS Institute, Cary NC).

## Results

Table 1 provides the distribution of state-level mortality outcomes and exposure to each risk factor by sex. Mean CV, CHD, and stroke mortality were each higher for men (316.5, 181.2, and 37.0 per 100,000, respectively) compared to women (162.7, 72.7, and 29.6 per 100,000, respectively) aged 45–74 y in 2010. Based on the relative standard deviations, CV and CHD mortality among women was more variable across states than among men. Regarding risk factors, elevated cholesterol and alcohol abstinence had the highest median prevalence for men and women, respectively, aged 40–69 y in 2005. Alcohol abstinence and diabetes were the most variable risk factors across states based on relative standard deviations.

**Table 1:** State-level CV risk index and risk factor prevalence for adults aged 40–69 y in 2005 and mortality rates aged 45–74 y in 2010 in the 50 US states and DC.

	Men			Women		
	Mean	SD	RSD	Mean	SD	RSD
Mortality rates (per 100,000)						
Cardiovascular	316.5	74.7	23.6	162.7	50.1	30.8
Coronary heart disease	181.2	39.8	22.0	72.7	25.1	34.6
Stroke	37.0	9.6	25.9	29.6	7.6	25.8
Risk factor prevalence, %						
Smoking	22.2	3.7	16.5	19.9	3.4	17.0
Obesity	29.2	3.6	12.5	28.2	3.9	14.0
Physical inactivity	24.0	4.4	18.3	26.3	5.4	20.5
Alcohol abstinence	41.6	9.9	23.9	54.2	10.9	20.1
Hypertension	34.7	4.2	12.1	31.8	4.7	14.8
Elevated cholesterol	44.4	3.0	6.7	39.1	3.0	7.7
Diabetes	10.8	2.2	20.4	9.5	2.3	24.6

CV, cardiovascular; RSD, relative standard deviation (100× standard deviation/mean).

Notes: Mortality rates are based on 2010 vital statistics data, and risk factor prevalence is based on 2005 BRFSS data. Mortality rates and risk factor prevalences were age- and sex- standardized to the 2010 US population. Because the CV risk index has a mean of 0, its RSD is undefined.

Correlations between state prevalence of individual risk factors and the CV risk index summarizing all seven risk factors are shown in Table S1 in File S1. With the exception of elevated cholesterol among men, all correlations between risk factor prevalence and the final CV index were statistically significant (p<.05) and ranged from 0.34 to 0.87 for men and 0.69 to 0.92 for women. To provide intuition regarding the interpretation of the CV risk index, Table S3 in File S1 shows the mean of state-level risk factor prevalence within quintiles of the CV risk index. As expected, the states with lower values for the CV risk index had lower mean prevalence of risk factors for both men and women. For example, the mean prevalence of male smoking was 19.0% for states in the lowest CV index quintile, compared to 26.4% for states in the highest CV index quintile. Overall, the state CV risk index summarized 56% and 69% of the variance contained in the original 7 risk indicators for men and women, respectively (data not shown). The Cronbach's alpha was 0.85 and 0.92 for the CV risk index for men and women, respectively, indicating that the behaviors were highly positively correlated with each other at the state-level and appropriately measured using a composite CV risk index.

**Figure 1:**
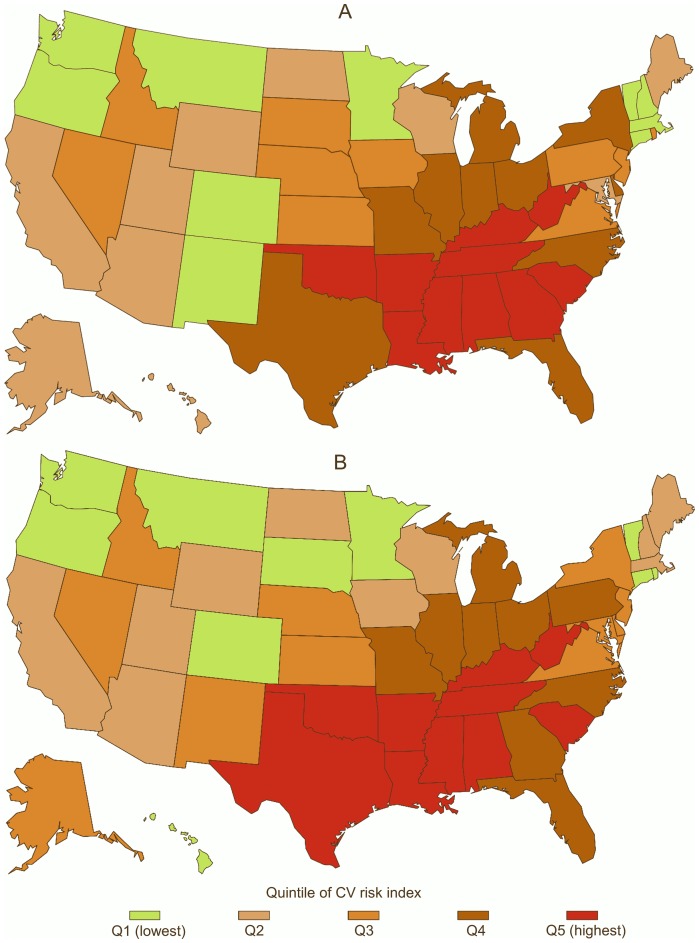
Geographical distribution of quintiles of state-level CV risk index among men (panel A) and women (panel B) aged 40–69 y in 2005 in the US. The CV risk index is a composite measure computed from principal components analysis of state-level risk factor prevalence data.

Figure 1 shows the geographical distribution of the CV risk index across the US. States in the highest CV risk index quintile were concentrated in the southeastern US, and states in increasingly lower quintiles tended to be distributed westward and northward. This pattern was consistent for men and women.

Table 2 reports correlations between state-level risk factors and mortality rates. All correlations were positive and statistically significant except for those between cholesterol and mortality among men. The highest correlations were generally observed for the CV risk index and mortality; these ranged from 0.77 to 0.87 among men and 0.77 to 0.89 among women.

**Table 2:** Bivariate Pearson correlations of 2005 state-level CV risk index and risk factor prevalence for adults aged 40–69 y with 2010 cause-specific mortality rates for adults aged 45–74 y.

	Mortality rates (per 100,000)					
	Men			Women		
	CV	CHD	Stroke	CV	CHD	Stroke
Risk factor prevalence, %						
Smoking	0.72*	0.67*	0.65*	0.73*	0.72*	0.62*
Obesity	0.51*	0.46*	0.50*	0.76*	0.70*	0.72*
Physical inactivity	0.74*	0.74*	0.65*	0.83*	0.76*	0.76*
Alcohol abstinence	0.61*	0.50*	0.60*	0.68*	0.54*	0.74*
Hypertension	0.87*	0.75*	0.85*	0.86*	0.72*	0.86*
Elevated cholesterol	0.23	0.13	0.15	0.48*	0.34*	0.52*
Diabetes	0.72*	0.58*	0.70*	0.82*	0.69*	0.78*
CV risk index	0.87*	0.77*	0.82*	0.89*	0.77*	0.86*

CHD, coronary heart disease; CV, cardiovascular.

*p<.05.

Table 3 presents the estimated the number of deaths per 100,000 associated with a SD difference in the CV risk index. In the unadjusted model for the 50 US states and DC, the CV mortality rate was higher by 65.3 (95%CI = 55.2,75.4) and 45.0 (95%CI = 38.8,51.2) deaths per 100,000 for men and women, respectively, for each +1 standard deviation difference in the CV risk index. The association between the CV risk index and CV mortality was attenuated but remained statistically significant after adjusting for state-level confounders (41.0 [95%CI = 26.3, 55.7] and 33.3 [95%CI = 24.4, 42.2] deaths per 100,000 for men and women, respectively). Similarly, the CV risk index was significantly associated with CHD and stroke mortality in adjusted models.

**Table 3:** Estimated differences in cause-specific state mortality rates in 2010 for US adults aged 45–74 associated with each standard deviation in state CV risk index.

	CV		CHD		Stroke	
	Deaths per 100,000 (95% CI)	R^2^	Deaths per 100,000 (95% CI)	R^2^	Deaths per 100,000 (95% CI)	R^2^
	Men					
Unadjusted model	65.3 (55.2,75.4)	0.76	30.8 (23.7,37.9)	0.60	7.9 (6.4,9.4)	0.68
Adjusted model	41.0 (26.3,55.7)	0.08	23.3 (11.0,35.6)	0.09	3.4 (1.3,5.5)	0.03
	Women					
Unadjusted model	45.0 (38.8,51.2)	0.80	19.5 (15.1,23.9)	0.60	6.6 (5.5,7.7)	0.75
Adjusted model	33.3 (24.4,42.2)	0.11	16.1 (8.6,23.6)	0.10	5.0 (3.0,7.0)	0.11

CHD, coronary heart disease; CV, cardiovascular; SD, standard deviation.

Notes: Unadjusted and adjusted associations were modeled using linear regression; mortality rates were per 100,000, age and sex standardized to the 2010 population. The unadjusted model included only the state CV risk index. The adjusted model included the following state-level variables in addition to the CV risk index: median income in 2010, mean percent of population living under the poverty line in 2004, mean county-level proportion Hispanic, mean county-level proportion black, proportion of counties in which >65% of residents aged 25–64 years neither received a high school diploma nor GED in 2000, proportion of counties in which <65% of residents aged 25–64 years were employed in 2000, proportion insured in 2005, and mean county-level number of medical specialists per 100,000. The semi-partial R^2^ for the CV risk index is reported for adjusted models. The semi-partial R^2^ for the CV risk index is reported for adjusted models.

The proportion of variation in CV mortality rates that could be attributed to the CV risk index ranged from 0.60 to 0.80 based on R^2^ values in unadjusted models and 0.03 to 0.11 based on semi-partial R^2^ values in adjusted models (Table 3). We observed the same pattern of results when the CV risk index was constructed using only the behavioral risk factors (i.e., smoking, physical inactivity, alcohol abstinence, and obesity; data not shown).

Tables S4–S6 in File S1 present results from sensitivity analyses. Overall, the sensitivity results supported the robustness of findings reported in Table 3. The adjusted analysis of non-stroke belt states after excluding stroke-belt states yielded results consistent with those observed for all 50 US states and DC (Table S4 in File S1). The CV risk index was not significantly associated with mortality from transport accidents or infant death rates after adjustment (Table S5 in File S1). County-level estimates were similar to those at the state level (Table S6 in File S1). In addition, after eliminating counties with the 10% highest levels of the CV risk index and highest levels of CV mortality, separately, we found similar associations to those estimated among all counties.

## Discussion

Our findings indicate that leading modifiable CV risk factors continue to aggregate at the state-level and are collectively associated with state-level variation in CV mortality. After accounting for differences in state compositional characteristics, a 1 SD higher CV risk index summarizing seven leading risk factors was associated with 41.0 (men) and 33.3 (women) more deaths per 100,000 in US states in 2010. The CV risk index independently explained roughly one-tenth of the variation in CV and CHD mortality for both men and women. We also note that socioeconomic, demographic, and health service characteristics of states explained a larger share of CV mortality variation across states than CV risk factors.

Comparing these findings to an earlier investigation by Byers et al using a similar study design and data sources, we found that the magnitudes of bivariate correlations between the state-level prevalence of hypertension and diabetes with CHD mortality were higher in the present analysis than in the early 1990s [10]. This may indicate a true change in the correlation. Alternatively, we used a lagged design while Byers et al used contemporaneous measures of risk and mortality. Consistent with a recent study using Framingham risk scores to assess state-level risk [4], we found that behavioral risk factors were more informative for explaining state variation in CHD mortality than stroke mortality among men.

Correlations between the risk factors and the final CV risk index suggest that these risk factors were highly correlated at the state-level in 2005, as they were nearly a decade prior [9]. Thus, some states face a disproportionate burden of multiple CV risk factors. We also demonstrated that the geographical distribution of the CV risk index mirrors the geographical distribution of mortality rates, such that both adverse CV behaviors and mortality are concentrated in the Southeast. Interestingly, however, the associations we report were also observed after excluding the stroke-belt states in the Southeast. State environments may contribute to unhealthy patterning of CV risk among residents; conversely, states are in a position to support residents in achieving healthier CV risk profiles. Previous CV mortality reductions in the US have been achieved in part through reductions in risk factors [11], [17], and the present analysis suggests that some states may stand to gain more than others from risk reduction.

Our analysis had both strengths and limitations. We generated a composite CV risk index to summarize state exposure to combined risks and subsequently examine the simultaneous influence of multiple CV risk factors on state disparities in mortality. Investigating CV risk factors simultaneously may be more valid than single-risk factor analyses that exclude correlated risk factors because of statistical collinearity. Using principal components analysis to generate the CV risk index was both novel and specifically suited for dealing with correlated indicators, such as these risk factors. The BRFSS provided the best available data to study state-level variation in CV risk because the survey was designed to be representative at the state-level with national coverage, allowing us to compare all US states and maintain high external validity. Similarly, we ascertained state-level mortality using vital statistics data, which were both nationally comprehensive and have low measurement error.

This study was ecological in design and we did not examine individual-level associations. All risk factors were based on respondent reports and subject to measurement error. This error may have resulted in conservative or inflated estimates of the associations between CV risk factors and mortality rates. While risk factors such as smoking, obesity, diabetes, and hypertension have been shown to be valid [18]–[20], concerns remain about the under-reporting of elevated cholesterol in the BRFSS [18], [21]. This reporting error may have contributed to the low correlation of this variable with mortality rates. In addition, we did not measure the degree of risk reported by respondents (e.g., years of smoking or years lived with diagnosis of diabetes) or incorporate sampling uncertainty into the index. We addressed key potential confounders including state-level sex, age, racial and economic composition, although it is possible that our results are confounded by other attributes of states. We additionally checked that associations were not solely attributable to the large differences in CV risk factors between stroke belt and non-stroke belt states. Finally, discriminant validation of the behavioral data was demonstrated by the lack of an association between the CV risk index and accident related mortality.

In sum, we found that states tend to possess uniformly high or low prevalence of risk factors. Many of the risk factors we examined share a similar set of behavioral antecedents—for example, hypertension and diabetes are both associated with obesity and smoking. Therefore, improving upstream behaviors may reduce multiple risk factors simultaneously. Reducing interstate CV mortality variation may also require addressing broader social and healthcare disparities, which our results indicated to be related to state-level CV mortality above and beyond risk factors.

## Supporting Information

Figure S1 The relation between the CV risk index and state-level cause-specific mortality rates in 2010 for men (top panel) and women (bottom panel) aged 45–74 y. Washington DC had relatively high CV mortality rates for its value of the CV risk index and appears to be an outlier; removing Washington DC from the analysis did not affect the analysis.
(TIF)

File S1 Supporting information containing Tables S1–S6.
(DOCX)
