# Supplementary material for: Interstate Variation in Modifiable Risk Factors and Cardiovascular Mortality in the United States
Source: PLoS One. 2014 Jul 8;9(7):e101531. doi: 10.1371/journal.pone.0101531 (PMC4086813; doi:10.1371/journal.pone.0101531)
Supplement: File S1 — Supporting information containing Tables S1–S6. (DOCX) [file pone.0101531.s002.docx]

**Table S1** Principal components loadings (correlation coefficients) between 2005 state-level risk factor prevalence for adults aged 40-69 y and the composite CV risk index

|  | Correlations with composite CV risk index  (factor loadings) | |
| --- | --- | --- |
| Prevalence of risk factor | Men | Women |
| Smoking | 0.75 | 0.73 |
| Obesity | 0.71 | 0.82 |
| Physical inactivity | 0.85 | 0.93 |
| Alcohol abstinence | 0.77 | 0.81 |
| Hypertension | 0.87 | 0.92 |
| Elevated cholesterol | 0.34 | 0.69 |
| Diabetes | 0.83 | 0.91 |

CV, cardiovascular

Notes: With the exception of elevated cholesterol among men, all p-values <.05 for testing correlations unequal to 0.

**Table S2** Principal components analysis scoring coefficients for each risk factor used to construct the composite CV risk index

|  |  |  |  |
| --- | --- | --- | --- |
|  | Males |  | Females |
| Smoking | 0.190 |  | 0.150 |
| Obesity | 0.182 |  | 0.168 |
| Physical inactivity | 0.217 |  | 0.190 |
| Alcohol abstinence | 0.195 |  | 0.167 |
| Hypertension | 0.221 |  | 0.189 |
| Elevated cholesterol | 0.085 |  | 0.142 |
| Diabetes | 0.211 |  | 0.186 |

**Table S3** Mean values of the 2005 state-level CV risk index and risk factor prevalence (%) for adults aged 40-69 y by quintiles of the CV risk index

|  | Men | | | | |  | Women | | | | |
| --- | --- | --- | --- | --- | --- | --- | --- | --- | --- | --- | --- |
|  | CV risk index quintile | | | | |  | CV risk index quintile | | | | |
|  | Lowest | Q2 | Q3 | Q4 | Highest |  | Lowest | Q2 | Q3 | Q4 | Highest |
|  | Mean | | | | |  | Mean | | | | |
| CV index score | -1.2 | -0.6 | -0.2 | 0.4 | 1.6 |  | -1.1 | -0.7 | -0.2 | 0.5 | 1.6 |
| Smoking | 19.0 | 20.4 | 21.4 | 23.8 | 26.4 |  | 17.3 | 17.4 | 19.7 | 22.0 | 23.4 |
| Obesity | 25.8 | 28.2 | 29.1 | 30.0 | 33.0 |  | 23.7 | 26.5 | 27.4 | 30.7 | 32.9 |
| Physical inactivity | 19.5 | 21.2 | 24.4 | 24.9 | 30.1 |  | 20.6 | 22.5 | 25.6 | 28.4 | 34.5 |
| Alcohol abstinence | 31.4 | 38.5 | 37.1 | 40.5 | 54.1 |  | 44.4 | 48.1 | 49.8 | 53.6 | 69.1 |
| Hypertension | 31.0 | 31.9 | 33.7 | 36.0 | 41.2 |  | 27.5 | 28.2 | 30.9 | 34.0 | 38.7 |
| Elevated cholesterol | 41.8 | 43.8 | 45.4 | 45.8 | 45.0 |  | 36.4 | 37.9 | 38.7 | 40.4 | 42.3 |
| Diabetes | 8.7 | 9.5 | 10.1 | 12.3 | 13.5 |  | 7.0 | 8.1 | 9.1 | 10.6 | 12.9 |

CV, cardiovascular; Q, quintile

**Table S4** Estimated differences in cause-specific mortality rates in 2010 for U.S. adults aged 45-74 associated with each standard deviation in state CV risk index within stroke-belt (n=8) and non-stroke belt states and DC (n=43)

|  | CV | |  | CHD | |  | Stroke | |
| --- | --- | --- | --- | --- | --- | --- | --- | --- |
| Model | Deaths per 100,000  (95% CI) | R^2^ |  | Deaths per 100,000  (95% CI) | R^2^ |  | Deaths per 100,000  (95% CI) | R^2^ |
|  | Non Stroke-belt: Men | | | | | | | |
| Unadjusted | 55.5 (41.6,69.4) | 0.60 |  | 32.3 (23.2,41.4) | 0.54 |  | 5.3 (3.4,7.2) | 0.44 |
| Adjusted | 42.2 (26.8,57.6) | 0.15 |  | 21.4 (10.3,32.5) | 0.10 |  | 3.4 (1.0,5.8) | 0.07 |
|  | Non Stroke-belt: Women | | | | | | | |
| Unadjusted | 41.7 (33.3,50.1) | 0.70 |  | 21.5 (15.8,27.2) | 0.57 |  | 5.6 (4.2,7.0) | 0.60 |
| Adjusted | 32.1 (22.2,42.0) | 0.14 |  | 11.7 (4.5,18.9) | 0.06 |  | 5.2 (2.9,7.5) | 0.18 |

CHD, coronary heart disease; CV, cardiovascular; SD, standard deviation

Notes: The stroke-belt was defined as NC, SC, GA, AL, MS, TN, AR, LO, and non-stroke belt states were all other states and Washington DC. Unadjusted and adjusted associations were modeled using linear regression; mortality rates were per 100,000, age and sex standardized to the 2010 population. The adjusted model included median income and proportion of black residents at the state-level in addition to the CV risk index; the semi-partial R^2^ for the CV risk index is reported for that model.

**Table S5.** Negative controls: Associations between the CV risk index and non-CV deaths across US states

|  | Transport Accident Deaths per 100,000  (95% CI) | R^2^ | p-value |  | Infant Deaths per 1,000 Live Births  (95% CI) | R^2^ | p-value |
| --- | --- | --- | --- | --- | --- | --- | --- |
| Model | Men |  |  |  | Men |  |  |
| Unadjusted | 4.4 (2.4,6.4) | 0.27 | <.0001 |  | 0.9 (0.6,1.2) | 0.52 | <.0001 |
| Adjusted | 3.2 (-0.4,6.8) | 0.04 | 0.0930 |  | 0.3 (-0.1,0.7) | 0.02 | 0.1090 |
|  | Women |  |  |  | Women |  |  |
| Unadjusted | 1.0 (0.2,1.8) | 0.11 | 0.0212 |  | 0.9 (0.6,1.2) | 0.51 | <.0001 |
| Adjusted | 0.2 (-1.1,1.5) | 0.00 | 0.8118 |  | 0.3 (-0.1,0.7) | 0.02 | 0.1737 |

Notes: Unadjusted and adjusted associations were modeled using linear regression; mortality rates were per 100,000, age and sex standardized to the 2010 population. The unadjusted model included only the state CV risk index. The adjusted model included the following state-level variables in addition to the CV risk index: median income in 2010, mean percent of population living under the poverty line in 2004, mean county-level proportion Hispanic, mean county-level proportion black, proportion of counties in which > 65% of residents aged 25-64 years neither received a high school diploma nor GED in 2000, proportion of counties in which < 65% of residents aged 25-64 years were employed in 2000, proportion insured in 2005, and mean county-level number of medical specialists per 100,000. The semi-partial R^2^ for the CV risk index is reported for adjusted models.

**Table S6** Estimated differences in county CV mortality rates in 2005-2010 for US adults aged 45-74 associated with each standard deviation in county CV risk index

|  | All available BRFSS counties in 2005 (n=1279) |  |  | Counties in the 90^th^ percentile or below for CV mortality  (n=1125) |  |  | Counties in the 90^th^ percentile or below for CV risk index (n=1125) |  |
| --- | --- | --- | --- | --- | --- | --- | --- | --- |
| Model | CVD Deaths per 100,000  (95% CI) | R^2^ |  | CVD Deaths per 100,000  (95% CI) | R^2^ |  | CVD Deaths per 100,000  (95% CI) | R^2^ |
|  | Men | | | | | | | |
| Unadjusted | 60.1 (55.0,65.2) | 0.31 |  | 43.5 (39.1,47.9) | 0.25 |  | 55.8 (49.6,62.0) | 0.22 |
| Adjusted | 21.4 (17.1,25.7) | 0.03 |  | 20.8 (16.7,24.9) | 0.04 |  | 21.6 (16.7,26.5) | 0.03 |
|  | Women | | | | | | | |
| Unadjusted | 44.8 (42.1,47.5) | 0.47 |  | 33.8 (31.3,36.3) | 0.40 |  | 44.6 (41.3,47.9) | 0.40 |
| Adjusted | 21.6 (18.6,24.6) | 0.06 |  | 20.2 (17.4,23.0) | 0.09 |  | 20.5 (17.2,23.8) | 0.05 |

Notes: Unadjusted and adjusted associations were modeled using linear regression; mortality rates were per 100,000 for 2005-2010. The unadjusted model included only the county CV risk index. The adjusted model included county-level proportion insured, mean number of medical specialists per 100,000, proportion Hispanic, proportion black, proportion of counties in which > 65% of residents aged 25-64 years neither received a high school diploma nor GED in 2000, percent of individuals living under the poverty line in 2004, and proportion of counties in which < 65% of residents aged 25-64 years were employed in 2000, in addition to the CV risk index. The semi-partial R^2^ for the CV risk index is reported for adjusted models.
